# Decomposition and entomological colonization of charred bodies – a pilot study

**DOI:** 10.3325/cmj.2013.54.387

**Published:** 2013-08

**Authors:** Stefano Vanin, Emma Zanotti, Daniele Gibelli, Anna Taborelli, Salvatore Andreola, Cristina Cattaneo

**Affiliations:** 1 School of Applied Sciences, University of Huddersfield, Queensgate, Huddersfield, UK; 2 LABANOF, Laboratory of Forensic Anthropology and Odontology, Legal Medicine, Department of Human Morphology and Biomedical Sciences, University of Milan, Milan, Italy

## Abstract

**Aim:**

To use forensic entomological approach to estimate the post mortem interval (PMI) in burnt remains.

**Methods:**

Two experiments were performed in a field in the outskirts of Milan, in winter and summer 2007. Four 60-kg pigs were used: two for each experiment. One pig carcass was burnt until it reached the level 2-3 of the Glassman-Crow scale and the not-burnt carcass was used as a control. In order to describe the decomposition process and to collect the data useful for minimum PMI estimation, macroscopic, histological, and entomological analyses were performed.

**Results:**

In the winter part of the experiment, the first insect activity on the burnt carcass began in the third week (*Calliphora vomitoria*) and at the beginning of the fourth week an increase in the number of species was observed. In the summer part, adult flies and first instar maggots (*Phormia regina*) appeared a few minutes/hours after the carcass exposure. Both in winter and summer, flies belonging to the first colonization wave (Calliphoridae) appeared on burnt and control pigs at the same time, whereas other species (Diptera and Coleoptera) appeared earlier on burnt pigs.

**Conclusion:**

In forensic practice, burnt bodies are among the most neglected fields of entomological research, since they are supposed to be an inadequate substratum for insect colonization. Entomological approach for PMI estimation proved to be useful, although further studies on larger samples are needed.

Estimation of the post mortem interval (PMI) plays an important role in forensic investigation. In the early post mortem period, PMI can be estimated by temperature-based methods, but when decomposition begins, this estimation can be influenced by several variables (1,2). In addition, in cases of concealment, body dismemberment, explosion, and burning there is no standardized method based on experimental studies for deriving time since death from morphological characteristics of the corpse.

Entomological approach is a well known and widely accepted method to estimate the minimum PMI (3). However, in the literature there are only a few cases referring to charred bodies (4-8). Gruenthal et al (9) found dung fly *Scathophaga stercoraria*, larvae of *Calliphora vicina* and *Calliphora vomitoria*, and immature beetle forms, not further identified in 24 pig carcasses charred up to Glassman Crow scale-1 (GCS-1) for the head, neck, limbs, and CGS 2 for the torso. Catts and Goff (6) observed a few days’ delay in the arrival of blowflies on a corpse burnt and charred inside an open-topped metal drum, and a week’s delay in the case of a pig burnt inside a car that was set afire (6). Introna et al (4) also highlighted that burnt flesh delayed the arrival of blowflies. Due to the relevant lack of literature, the aim of our study was to report the results of an experimental approach to burnt bodies using pigs (*Sus scrofa*) as models.

## Material and methods

Two experiments were performed in a field in the outskirts of Milan, in Northern Italy (45° 20’ N; 09 13’ E), in the winter and summer of 2007. The weather at the site was hot and damp in the summer and cold in the winter with moderate surface winds. Meteorological data were collected from the closest meteorological station located 1.5 km from the studied area and compared with the measurements performed during the sampling.

Adult domestic pig (*Sus scrofa*) carcasses were used as models for human cadavers. This animal is considered to be an excellent model for human decomposition and is frequently used in taphonomic experiments, particularly concerning insect/arthropod colonization (6,10-18).

Four 60-kg pigs were obtained from the Department of Veterinary Medicine (University of Milan); each animal died from causes independent from the experimental project. For each experiment, a pig carcass was burnt on a wooden pyre until it reached the level 2-3 of the Glassman-Crow scale (19), corresponding to the destruction of the extremities, initial charring of the skin, and a substantial preservation of the corpse. The second pig of similar weight, not burnt, was used as control. In both experiments, the carcasses were maintained at a 50 m distance from each other to avoid reciprocal contamination and a wire mesh (5 cm mesh size) was placed over each carcass to prevent vertebrate depredation. The animals were placed in the same place during the winter and summer experiments.

Observation and sample collections were performed after 3, 6, 15, 18, 24, 36, 42, 60, 95, and 120 days in the winter and after 1, 6, 9, 12, 15, 18, 27, 34, and 42 days in the summer. For each observation, carcasses were macroscopically analyzed in order to determine the state of decomposition according to the Goff terrestrial model, which distinguishes fresh, bloated, decay, postdecay, and skeletal stages (20).

In order to perform the morphological evaluation of the exposed pigs, samples for histological analyses (square in shape, 1 cm wide) were taken from the charred skin areas, and fixed in 10% formalin and then paraffin-embedded according to standard histological technique. Four microtome-thick sections were cut from paraffin blocks and stained with standard hematoxylin eosin stain and Trichrome stain. All observations were made using a light microscope equipped with a digital camera and DP software for computer-assisted image acquirement and managing (Wild Heerbrugg, Switzerland).

Eight insect pitfall traps containing a saturated NaCl solution and soap were placed at 50 cm all around the carcass. Moreover, entomological samples were collected by hand on the carcasses, under them, and where and when possible in the carrion cavities. Insect identification was performed using specific entomological key and description (21-24) and by comparison with specimens stored in the private collection of one of the authors (SV). Zoological nomenclature followed Minelli et al (25).

## Results

The first part of the study was conducted from February to June. The average temperature in this period was 16.0°C (min 1.0°C, max 32.4°C) and the rainfall was considerable during March (48 mm) and May (152 mm). The second part of the study was conducted from June to August. The average temperature was 25.5°C (min 12.8°C, max 36.0°C). The rainfall during this time was negligible.

**Figure 1:**
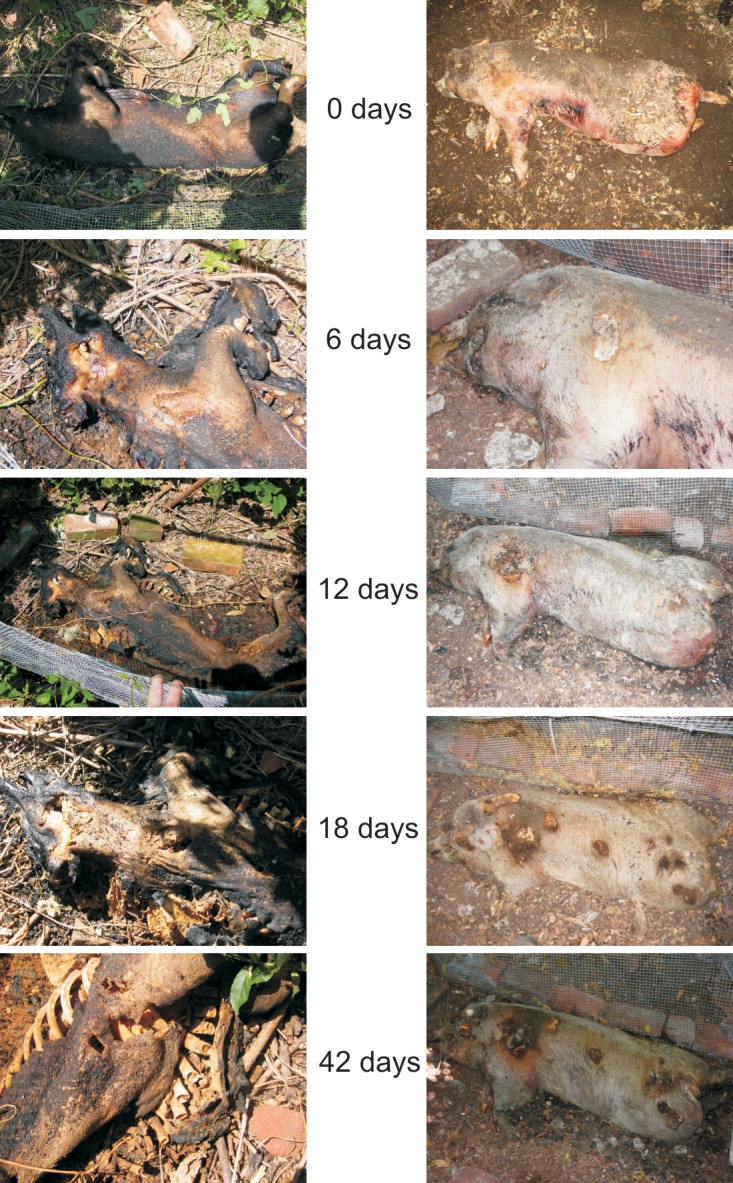
Experiment performed during the winter: stages of decomposition of the burnt (left) and the control (right) pigs.

**Figure 2:**
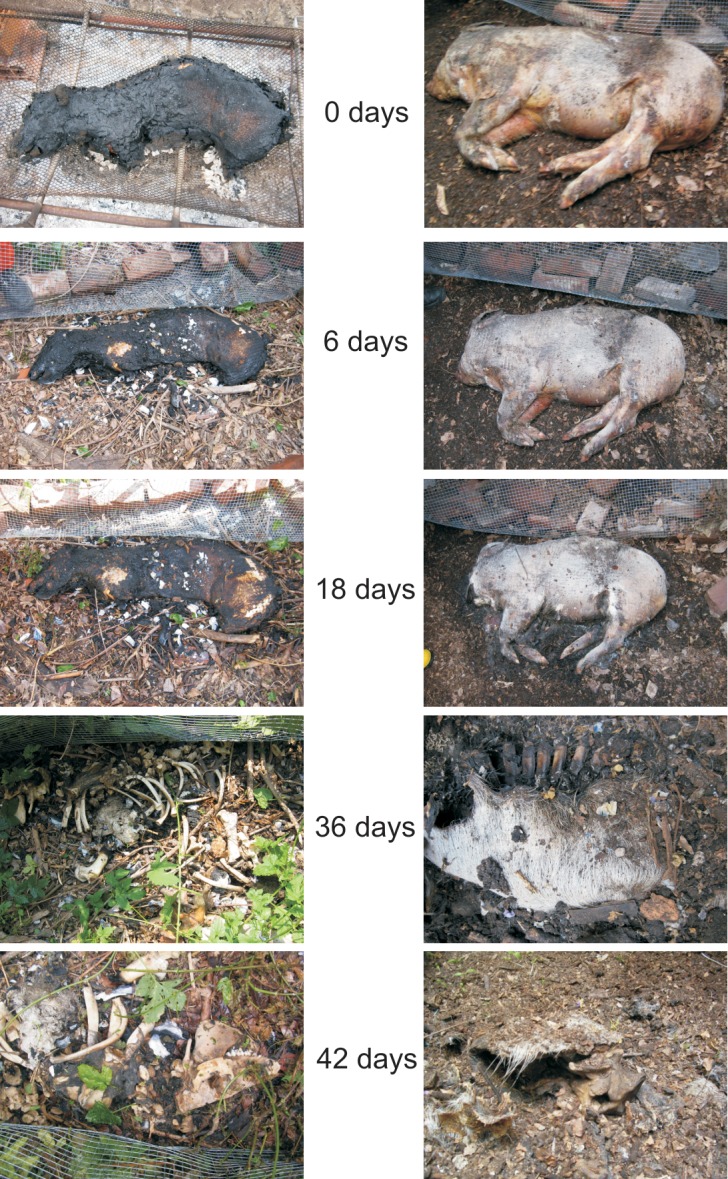
Experiment performed during the summer: stages of decomposition of the burnt (left) and the control (right) pigs.

Macroscopic (Figure 1 and Figure 2), histological, and entomological observations were carried out in order to describe the decomposition processes and insect colonization. The list of the saprophagous and saprophilous insects collected on the carcasses is shown in Table 1.

**Table 1:** Saprophagous and saprophilous species collected during the experiments.* In Diptera (^D^) the presence of larvae is reported and in Coleoptera (^C^) the presence of adults.

	Control							Burnt						
	Winter				Summer			Winter				Summer		
	II	III	IV	V	VI	VII	VIII	II	III	IV	V	VI	VII	VIII
*Calliphora vomitoria* ^D^														
*Calliphora vicina* ^D^														
*Phormia regina* ^D^														
*Lucilia sericata* ^D^														
*Chrysomya albiceps* ^D^														
*Sarcophaga* sp ^D^														
*Ophira capensis* ^D^														
*Fannia* sp ^D^														
*Sepsis* sp ^D^														
*Themira* sp ^D^														
*Sphaerocera curvipes* ^D^														
*Creophilus maxillosus* ^C^														
*Trox* sp ^C^														
*Thanatophilus sinuatus* ^C^														
*Silpha tristis* ^C^														
*Necrodes littoralis* ^C^														
*Abax ater inferior* ^C^														
*Paecilus cupreus* ^C^														
*Pseudophonus rufipes* ^C^														
*Aphodius* sp ^C^														
*Dermestes laniarius* ^C^														
*Dermestes frischii* ^C^														
*Margarinotus brunneus* ^C^														
*Saprinus caerulescens* ^C^														
*Saprinus semistriatus* ^C^														
*Saprinus subnitescens* ^C^														
*Saprinus tenuistriatus* ^C^														
*Omosita colon* ^C^														
*Nitidula carnaria*														

*Several species of Staphylinidae was collected (*Aleochara* cfr *bipustulata, Aleochara curtula, Aleochara gregaria, Aleochara intricata, Anotylus nitidulus, Astrapacus ulmi, Atheta laticollis, Atheta longicornis, Ontholestes murinus, Philontus concinnus, Platydracus stercorarius*) in one or two specimens, only *Creophilus maxillosus* was collected in several specimens.

During the first two weeks of the winter part of the experiment, after the charring process, no clear external modifications occurred on the carrion, there was no decomposition fluid, and no insect egg depositions. The first insect activity (flight) (*Calliphora vomitoria*) was observed at the day 18, but without egg laying. During the fourth week (day 26), a considerable presence of calliphorid maggots (*C. vomitoria*) and several adults and larvae of other Diptera [*Sphaerocera curvipes* (Sphaeroceridae), *Themira* sp, *Sepsis* sp (Sepsidae), Gen. spp (Sciaridae)] and Coleoptera (Staphylinidae, mainly *Creophilus maxillosus*) was recorded. At the same time, a clear reduction of the tissues in several body regions (head, thorax, and abdomen) was observed. On the day 26, the carrion appeared completely skeletonized, with complete bone disarticulation. The maggot activity (*C. vomitora, Calliphora vicina, Phormia regina, Hydrotaea capensis*) was localized only on the soil and under a few skin fragments, whereas Coleoptera (Silphidae, Staphylinidae, Carabidae, Anthicidae) were widely spread on all the body remains. There was no presence of larvae from the sixth week after exposure (day 42 and the following days). Two months after the exposure (day 60), the bones were clean and only a few remains of burnt skin and muscles were still present. Larvae and adults of coleopterans belonging to different families (Staphylinidae, Carabidae, Trogidae, and Aphodidae) were still recovered.

The control pig was exposed at the same time as the burnt pig. No evident morphological modifications were observed in the first 15 days after the exposure. The beginning of the initial putrefactive stage was detected at the end of the second week. In the third week (day 18), the presence of first instar larvae (*C. vicina*), adults of scuttle flies and Coleoptera (Staphylinidae, Carabidae, Trogidae) was recorded. At the same time, a moderate emphysematic phase in the head region and discharge of decomposition fluids from the mouth was observed. In the abdominal region, the beginning of a colliquative phase was observed. With the progression of this phase, breaking of the skin was evident and the maggot activity (*C. vicina, C. vomitoria, Ph. regina, H. capensis, Themira* sp) was localized mainly in the bodily area in contact with the soil. The rest of the skin became drier and drier and the maggot mass invaded the whole abdominal and thoracic cavities.

In the summer period, in the days immediately after the exposure the burnt carrion showed important morphological modifications, with evident decomposition processes in the head and the presence of adult flies and first instar maggots (*Ph. regina*) concentrated on the head and in the abdominal and thoracic cavities. The entry of the larvae into the body cavities occurred through the skin fissures caused by fire. After one week (day 6), the carrion showed some clear skeletonized areas (head, thorax). After the first week, the rate of skeletonization and the exposure of bones slowed down. Larval activity (*Ph. regina, Lucilia sericata*) was concentrated only under the skin, whereas coleopterans of different families (Staphylinidae, Carabidae, Silphidae, Histeridae, Anthicidae) were largely spread. During the third week (day 18), no maggots were observed on the body. In the fourth week (day 27), soft tissues were almost completely lost, except for large fragments of dry or burnt skin; a decrease in species richness was observed. Several Dermestidae larvae (*Dermestes laniarius*, *Dermestes frishii*) were present in all the body regions. In the sixth week, all the bones were exposed, with disarticulation of leg bones.

The control pig in the summer period was exposed on the same day as the burnt one. The decomposition processes during the next 2 months followed the typical pattern with the bloated, active, and dry decay stage and the beginning of the skeletonization phase. The most important concentration of maggots (*Ph. regina, L. sericata*) was detected in the abdominal area. After 6 weeks, the control pig showed about 40% skeletonization. Coleopterans (Staphylinidae, Carabidae, Silphidae, Histeridae, Anthicidae, Dermestidae) were collected during the whole decomposition period.

**Figure 3:**
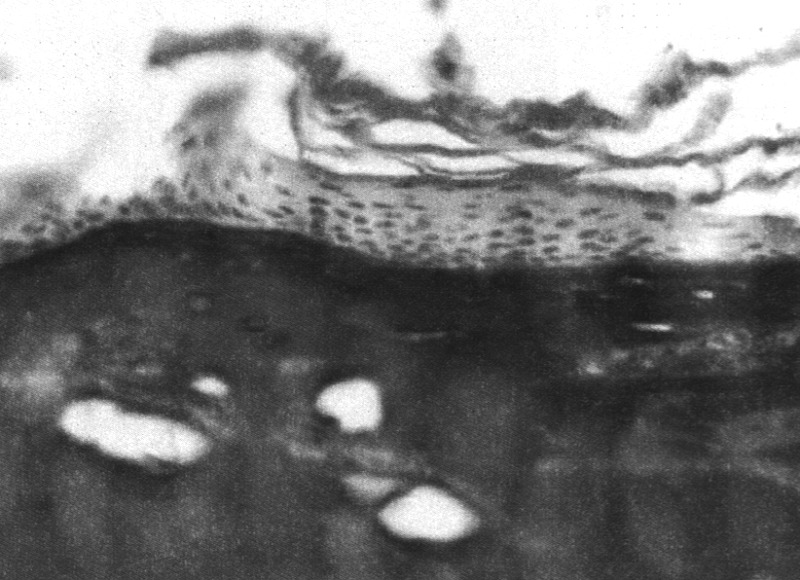
Histological section of a sample of charred skin stained with hematoxylin-eosin stain. Ovoidal, picnotic nuclei are visible; the derma is easily recognizable by the effect of protein clotting ( × 400).

Histological screening of the charred skin fragments showed that the outer crisp surface was completely destroyed macroscopically; but frequently underneath there was a thin layer of dehydrated skin, which showed moderate preservation of cellular patterns. The dermis, subcutaneous adipose, and muscle tissues were always visible during the whole experiment, both in the winter and summer part (Figure 3).

## Discussion

The entomo-fauna that was collected during the two experiments in both not-burnt and burnt carcasses included a large number of species typical of all colonization waves (23). The first flies (*Calliphora* spp, *Ph. regina*, *L. sericata*) arrived on burnt and not-burnt carcasses at the same time, whereas other species (Diptera and Coleoptera) arrived earlier on burnt carcasses. The colonization of burnt carcasses showed a classic pattern for the first wave insects and anticipation of other waves, with insects usually attributed to different waves arriving at the same time. This was probably a result of the carbonization process, which brought about two main advantages for fly colonization; first, the breaking up of charred soft tissues, with multiple fissures of the skin exposing the viscera, which therefore become immediately available for fly colonization. This may explain why the flies attacked the charred carcass earlier than the control carcass. In addition, the disruption of the skin surface and fast decomposition of the viscera during the first days creates a substratum of tissues in different phases of decomposition and with different water concentrations. This complex environment may explain the arrival of different waves of insects within a short period of time, which in cases of standard decomposition usually arrive sequentially. Moreover, the burning process caused the transformation of several molecules resulting in a wide spectrum of odors (volatile molecules), attractive for different insects.

Entomological approach proves to be a reliable method for the time since death determination. PMI estimation is of utmost importance in cases involving charred corpses, where decomposition processes often have different dynamics, and tissue destruction prevents evaluation based on morphological appearance or chemical variation, although some studies on accumulated degree days produced useful results (9,26). Some authors recently calculated a correction factor of the original formula, which adequately takes into account the carbonization process, but at the moment the morphological approach for PMI estimation is experimental, and there are still doubts concerning the standardization of specific carbonization variables (temperature, use of accelerants, etc) (27).

In this study, decomposition of burnt carcasses stopped quite soon, which is in contrast with four stages of decomposition observed by Avila and Goff (7). Both in winter and summer, charred tissues did not show what could be referred to as actual decomposition; they became more brittle and were affected by progressive crumpling, which lasted during the entire experimental period, reducing the bodily area covered by the soft tissues and exposing the bone surface (7). Charred corpses were better conserved probably due to the loss of water, with a decrease in bacterial activity, which was confirmed by histological analysis showing that heat retains the tissues, probably through dehydration. In fact, the observed coartation of the dermis and the presence of large gas bubbles in the dermis and picnotic nuclei in the epithelium are reported to be changes caused by heat (28-35). The results are in contrast with data reported by Gruenthal et al (9), which verified the decomposition rate in charred pig carcasses, and observed that, although the general decomposition trend was similar both in charred and uncharred carcasses, a more advanced pattern was visible in bodily regions highly affected by fire (9). The differences in the decomposition process found in these two studies may be explained by the lower carbonization degree and a limited charred area in the study by Gruenthal et al (9).

In conclusion, our results showed that in burnt remains, entomological approach can be used for estimation of the minimum PMI in the presence of insects belonging to the first colonization wave (mainly Calliphoridae). Our study does not support the claim that the burnt corpse is hardly colonized by flies (3,6) and indicates that the “classic” insect waves of colonization model reported by several authors cannot be applied to burnt remains. Further research is needed to evaluate the influence of temperature of carbonization, accelerants use, environment, and body size in PMI estimation in charred bodies and fill in the gap in this important field of forensic practice.
